# Effectiveness of Plant Beneficial Microbes: Overview of the Methodological Approaches for the Assessment of Root Colonization and Persistence

**DOI:** 10.3389/fpls.2020.00006

**Published:** 2020-01-31

**Authors:** Ida Romano, Valeria Ventorino, Olimpia Pepe

**Affiliations:** ^1^ Department of Agricultural Sciences, University of Naples Federico II, Naples, Italy; ^2^ Task Force on Microbiome Studies, University of Naples Federico II, Naples, Italy

**Keywords:** bioinoculant, plant growth-promoting microbes, colonization, persistence, culture-dependent methods, microscopy-based techniques, metagenomic approach

## Abstract

Issues concerning the use of harmful chemical fertilizers and pesticides that have large negative impacts on environmental and human health have generated increasing interest in the use of beneficial microorganisms for the development of sustainable agri-food systems. A successful microbial inoculant has to colonize the root system, establish a positive interaction and persist in the environment in competition with native microorganisms living in the soil through rhizocompetence traits. Currently, several approaches based on culture-dependent, microscopic and molecular methods have been developed to follow bioinoculants in the soil and plant surface over time. Although culture-dependent methods are commonly used to estimate the persistence of bioinoculants, it is difficult to differentiate inoculated organisms from native populations based on morphological characteristics. Therefore, these methods should be used complementary to culture-independent approaches. Microscopy-based techniques (bright-field, electron and fluorescence microscopy) allow to obtain a picture of microbial colonization outside and inside plant tissues also at high resolution, but it is not possible to always distinguish living cells from dead cells by direct observation as well as distinguish bioinoculants from indigenous microbial populations living in soils. In addition, the development of metagenomic techniques, including the use of DNA probes, PCR-based methods, next-generation sequencing, whole-genome sequencing and pangenome methods, provides a complementary approach useful to understand plant–soil–microbe interactions. However, to ensure good results in microbiological analysis, the first fundamental prerequisite is correct soil sampling and sample preparation for the different methodological approaches that will be assayed. Here, we provide an overview of the advantages and limitations of the currently used methods and new methodological approaches that could be developed to assess the presence, plant colonization and soil persistence of bioinoculants in the rhizosphere. We further discuss the possibility of integrating multidisciplinary approaches to examine the variations in microbial communities after inoculation and to track the inoculated microbial strains.

## Introduction

The increasing demand to reduce the use of chemical fertilizers and pesticides for the development of an agri-food system sustainable for environmental and human health, as well as the current shifting in the agricultural legislation of several countries, have led to an expanded use of bioinoculants. Chemical inputs usually alter the natural physico-chemical and biological equilibrium of soil, and microbial consortia used in agricultural management practices could return soil to its natural status (Lucy et al., 2004; Woo and Pepe, 2018). Although the manipulation of soil microbiomes to optimize crop productivity is an ancient practice, it is still little explored, especially regarding mechanistic studies of plant–microbe interactions and microbial persistence in heterogeneous communities in diverse locations, soils, and hosts (Finkel et al., 2017). Among the numerous bacterial or fungal strains used as bioinoculants, plant growth-promoting microbes (PGPM) are the most commonly applied. PGPM may affect plant performance through multiple mechanisms of action, operating directly by the production of specific substances that are able to promote plant growth and increase the availability and uptake of nutrients in soil (i.e., phosphate solubilization, siderophore and indole-3-acetic acid production, nitrogen fixation) or indirectly through the suppression of plant pathogens (Ribeiro and Cardoso, 2012). Several plant growth-promoting rhizobacteria (PGPR) have also been demonstrated to exert a beneficial effect on plant growth under nutritional and abiotic stress (Sharma et al., 2014; Singh and Sharma, 2016; Van Oosten et al., 2018) or during the restoration of polluted soils (Ventorino et al., 2014). Moreover, plants could also establish symbiosis with arbuscular mycorrhizal fungi (AMF), which increase the root surface area for nutrient acquisition (Wu et al., 2005).

A successful microbial inoculant has to colonize the external and/or internal part of plant tissues and establish a compatible interaction with the host as well as to persist in the soil against autochthonous microorganisms living in environment through its rhizocompetence traits (Finkel et al., 2017). In general, rhizosphere colonization occurs through several different mechanisms, such as bacterial movement, survival in the rhizosphere by competition against other microbes, adherence to and colonization of root surfaces, for instance by biofilm formation, and the creation of synergistic interactions with the host plant (Bhattacharya et al., 2017). Moreover, even if PGP inoculants colonize the plant initially, their persistence over time is not guaranteed. Measuring the persistence of microbial inoculants in soil poses technical difficulties, as the inoculant needs to be identified from within a complex community. The tracking and monitoring of the persistence of PGPM released in the environment have been widely studied (Brandt and Kluepfel, 1991; Kloepper and Beauchamp, 1992; Stahl and Kane, 1992; Gamalero et al., 2003; Podile and Kishore, 2006; Ahmad et al., 2011; Glick, 2015; Rilling et al., 2019) to understand their behavior in soil and which factors influence their survival under various conditions. Several sets of techniques are currently used to detect root colonization and persistence in the soils: microbial enumerations by culture-based methods, microscopy-based techniques, and DNA-based methods. The results may depend on the choice of technique since each has advantages and limitations, and each technique may have bias in favor of specific microbial taxa.

This review examines and presents an overview of the current methodological approaches that could be used to assess and detect plant colonization and soil persistence of microbial bioinoculants in the rhizosphere environment and considers multidisciplinary approaches to track and monitor inoculated microorganisms.

## Good Practices for Rhizosphere Sampling and Soil Preparation

In natural ecosystems such as soils, several variables or factors can influence the results due to the highly heterogeneous distribution of microbial cells in the environment. Therefore, a well-organized experimental plan to investigate microbial populations from plant roots and soil is necessary. Usually, in field experiments, the simplest approach used to overcome spatial variables is a completely randomized design with replicates since the treatments are assigned completely at random, creating homogeneous treatment groups (Fiorentino et al., 2018; Lusiba et al., 2018).

To ensure good results in microbiological analysis, the first fundamental prerequisite is the correct soil sampling, both in laboratory and in greenhouse trials and in field experiments, to obtain representative samples for each treatment to be analyzed (Pennock et al., 2008). Temporal and spatial aspects could be considered during rhizosphere (soil area influenced by plant roots and their exudates; Barillot et al., 2013) or bulk soil (soil not adhering to roots and not influenced by exudates; Barillot et al., 2013) sampling since changes in microbial diversity over time are usually related to environmental changes. Therefore, soil or rhizosphere microbial diversity studies are usually carried out over years or seasons (Lombard et al., 2011). Moreover, it is known that other factors, such as plant age and developmental stage, could also influence plant microbial community structure (Compant et al., 2019); therefore, these variables could also be considered for soil sampling.

Soil and rhizosphere samples can be collected by different sampling approaches, as extensively detailed by Wollum (1994): i) simple random, which ensures that each sample has the same opportunity to be selected, usually by using a grid; ii) stratified random, similar to simple random, except the area to be sampled is broken into smaller subareas; or iii) systematic, which ensures that the entire area is sampled and represented by individual samples that are obtained by establishing predetermined points. The number of soil samples to take depends on the microbial population distribution and can be calculated using the formula suggested by Wollum (1994), which considers a prestudy sampling, the sample variance and the sample mean. However, it is recommended to brush away stone, rubbish, trash or grass from the soil surface before taking samples. Then, using a sanitized shovel, it is possible to take the samples from topsoil to an adequate depth (for instance, 0-20 cm) or to collect plant roots by excavating or uprooting plants to study microbial diversity in bulk soil and/or rhizosphere. For rhizosphere studies, after plant sampling, roots should be shaken vigorously by hand to remove bulk soil and to collect soil adhering to roots (Ventorino et al., 2012; Barillot et al., 2013). Moreover, during the sampling, it is necessary to avoid root damage. Manual excavation using spades and hand tools and working progressively in layers or sectors could minimize the corruption of soil architecture and ensure the safety of the roots. It is also fundamental to take a sufficient number of replications for data analysis (Neumann et al., 2009). Following this, the samples must be recovered in sterile polyethylene bags or vessels and stored at 4°C to avoid desiccation during transport to the laboratory.

**Figure 1:**
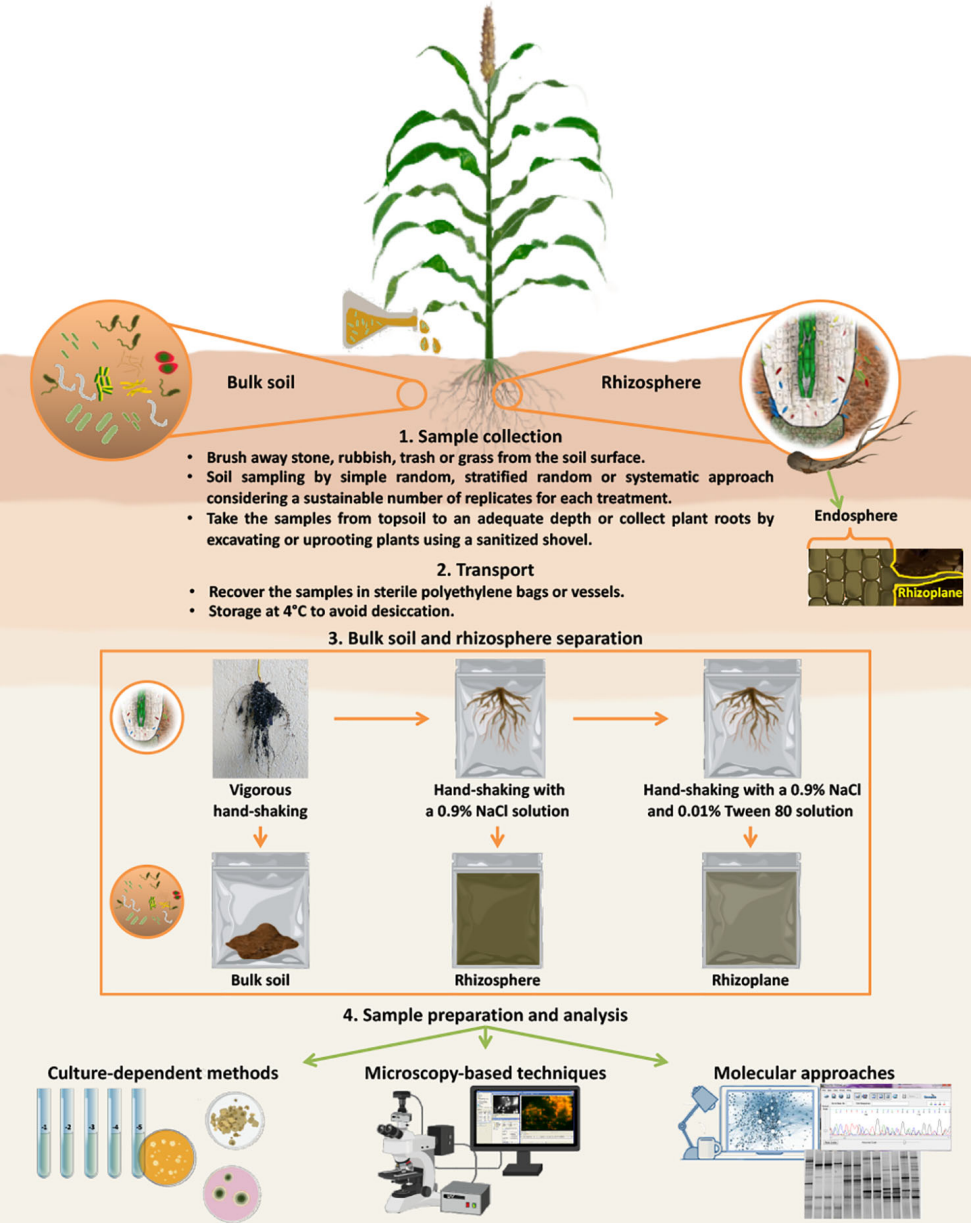
Schematic description of sampling collection, separation of different soil fractions, and methods (culture-dependent methods, microscopy-based techniques and molecular approaches) for the detection of microbial inoculants. After plant sampling, roots should be shaken vigorously by hand to collect bulk soil (soil not adhering to roots and not influenced by exudates). Shaking the roots a second time in a sterile 0.9% NaCl solution allowed rhizosphere (soil area influenced by plant roots and their exudates) collection, and shaking the roots a third time in the same sterile solution containing Tween 80 (0.01% v/v) allowed the rhizoplane (thin layer of soil strongly adhering to the roots) fraction to be collected. To study microbial endophytes, it is necessary to add a step of sterilization of the root surfaces prior to grinding, chopping or blending them. Root samples should be analyzed in a short time (24–48 h) to evaluate the density of the cultivable microorganisms by plating on growth media or they can be stored in a solution PBS buffer and 70% ethanol at -20°C for later analysis by culture-independent methods (microscopic and molecular methods).

To evaluate external and internal root colonization, which generally occur in the rhizoplane and endosphere, respectively, several steps for sample preparation are necessary (**Figure 1**). In particular, plant roots should be washed by agitation in sterile water or buffer [e.g., phosphate buffered saline (PBS) or physiological buffers] without tearing or cutting plant tissues to facilitate the separation between soil/root particles and microorganisms (Kloepper and Beauchamp, 1992). For instance, a good practice to detach the bacteria from the soil particles is shaking for 30 min at 120-130 rpm in an adequate volume of isotonic solution containing tetrasodium pyrophosphate (16% w/v) (Ventorino et al., 2014). Barillot et al. (2013) reported that after vigorously hand-shaking roots to separate bulk soil from rhizospheric soil, shaking the roots a second time in a sterile 0.9% NaCl solution allowed rhizosphere collection, and shaking the roots a third time in the same sterile solution containing Tween 80 (0.01% v/v) allowed the rhizoplane fraction (thin layer of soil strongly adhering to the roots; Barillot et al., 2013) to be collected (**Figure 1**). Indeed, to study microbial endophytes, it is necessary to surface sterilize the roots prior to grinding, chopping or blending them (McInroy and Kloepper, 1991). Several works describe a prior wash with 1% chloramine and cycles of washing/agitation treatments using ethanol and PBS (Ladha et al., 1997; Dennis et al., 2008; Richter-Heitmann et al., 2016). Cleaned roots to be analyzed by culture-independent methods can be stored in a solution of PBS buffer and 70% ethanol (2:3 v/v) for a long time at -20°C (Dennis et al., 2008; Richter-Heitmann et al., 2016). However, fresh root samples used to evaluate the density of the cultivable microorganisms by plating on growth media should be analyzed within a short time (24–48 h).

## Microbial Enumerations by Culture-Dependent Methods

Mainly because of their ease of use, culture-dependent methods are commonly used to estimate the persistence of inoculated microorganisms in soil and/or rhizosphere. However, these methods are limited since it is difficult to represent the high diversity of bacteria on culture media because only 0.1 to 1.0% of soil bacteria are cultivable (Daniel, 2005), and at the same time, it is difficult to differentiate inoculated organisms from native populations based on morphological characteristics (Lima et al., 2003).

To increase the likelihood of cultivating a high number of microbial strains, enrichment, selective and differential media are usually used as well as synthetic media mimicking the soil environment, typically containing soil extracts, are also developed. This approach has been successful, and it allowed the detection of a higher diversity of cultivable populations compared with other methods (Andreote et al., 2009). Although culture-dependent methods have been used to detect bioinoculants in different experimental conditions (growth chamber, greenhouse, open field), they are especially useful when the experiment is carried out in sterile conditions and interference by soil autochthonous microbial populations can be avoided. Therefore, advantages and limitations of culture-dependent approaches will be discussed on the basis of experimental conditions (i.e. growth chamber, greenhouse, field).

### Growth Chamber

Experiments conducted in growth chambers are usually performed using sterile synthetic substrates or hydroponic conditions for plant growth, allowing the control of all environmental parameters, such as temperature, relative humidity, light/dark cycle, and light intensity. Therefore, this approach is particularly suitable for the detection of inoculated strains in plant tissues by enumeration on culture media.

Castanheira et al. (2017) used viable counts to assess the colonizing abilities of a bacterial consortium composed of *Pseudomonas* sp. G1Dc10, *Paenibacillus* sp. G3Ac9, and *Sphingomonas* (*S*.) *azotifigens* DSMZ 18530 on the rhizoplane and surface-disinfected roots, stems and leaves of annual ryegrass plants grown under gnotobiotic conditions (**Table 1**). Sterile experimental conditions allow the use of a unique generic growth substrate to perform total bacterial counts and can allow three different bacterial strains to be distinguished on the basis of colony morphology.

**Table 1:** Culture-dependent approach used to monitor plant growth-promoting bacteria and root interaction.

Strains	Experimental conditions	Microbial media	Plant substrate	Results	References
*Pseudomonas* sp. G1Dc10 *Paenibacillus* sp. G3Ac9 *Sphingomonas azotifigens* DSMZ18530	Gnotobiotic conditions in controlled-environment chamber (16-h light/8-h dark, 18–23°C)	TY agar	Modified Evans medium supplemented with 8% agar	Colonization density in the rhizoplane and in the leaves was about 9 and 4 log_10_ CFU/g, respectively. Colonization was more abundant in the rhizoplane than in plant tissues.	Castanheira et al., 2017
*Pseudomonas* sp. VM1449 *Pseudomonas* sp. VM1450 *Pseudomonas* sp. VM1453	Pots (16-h light/8-h dark, 20–25°C)	PCA containing 100 µg/ml kanamycin	Sterilized compost/vermiculite (3:1 ratio)	The three bacterial strains showed different colonization behavior (CFU/g) for rhizosphere, interior root tissues stems or leaves	Germaine et al., 2004
*Burkholderia* sp. WPB *Rhizobium tropici* PTD1 *Rahnella* sp. WP5	Axenic conditions in growth chamber	MG/L with 100 µg/ml of gentamycin and carbenicillin	N-free MS agar	Higher endophyte populations (CFU/g) were observed in the roots when compared with the stem and leaves	Kandel et al., 2015
*Azotobacter chroococcum* HKN-5 *Bacillus megaterium* HKP-2 *Bacillus mucilaginous* HKK-2 *Glomus mosseae* *Glomus intraradice*	Pots in greenhouse (20 ± 4°C; 87 days)	Specific media for N-fixing bacteria, P solubilizer and K solubilizer	Soil (pH 5.46, organic matter 1.08%, total N 0.062%, total K 7,408 mg/kg, total P 1,090 mg/kg)	The population size of the inoculated rhizobacteria varied in accordance with the levels of fertilization and AMF colonization in the rhizosphere	Wu et al., 2005
*Azotobacter chroococcum* *Bacillus megaterium* *Bacillus mucilaginous* *Glomus fasciculatum* *Glomus mosseae*	Greenhouse (21 ± 5°C; 45 days)	Differentiating media for N-fixing bacteria, P solubilizer and K solubilizer	Sterilized soil (pH 7.32, EC 0.14 dS/m, total C 1.92%, total N, 0.19%, total K 2,063 ppm)	Root colonization by AMF was increased in the presence of bacterial consortium application in comparison to individual inoculation treatments	Khalid et al., 2017
*Azotobacter* strain ST3 *Azotobacter* strain ST6 *Azotobacter* strain ST9 *Azotobacter* strain ST17 *Azotobacter* strain ST24	Pot house; sampling at 30, 60, and 90 days	Nutrient agar	Four different unsterilized saline soil	Survival of inoculated strains increased up to 60 days of sampling	Chaudhary et al., 2013
*Azotobacter chroococcum* 76A	Greenhouse (10 cm plastic pots)	LG agar	Pure peat moss under salt stress	The bacterial strain was able to grow in the rhizosphere of tomato plants under abiotic stress conditions increasing of 1 Log	Van Oosten et al., 2018
*Azotobacter chroococcum* Mac 27L	Pots; sampling after 30 and 60 days of growth	Burks medium plates with and without X-gal	Unsterilized soil	The bacterial strain was able to survive in the rhizoplane of *Brassica campestris* up to 30 days after sowing	Solanki and Garg, 2014
*Azotobacter chroococcum* AZ1 *Azotobacter chroococcum* AZ2 *Glomus mosseae* *Glomus fasciculatum*	Plots, temperate rainfed conditions	Nutrient agar medium, coal-vitamin medium, potato-dextrose supplemented with Rose-Bengal and streptomycin (30 g/ml)	Solarized, disinfected and natural soil plots (21% sand, 35.7% silt 43.3% clay; pH 7.4)	An increase of concentration of bacteria and/or fungal strains in the inoculated tests has been registered	Sharma et al., 2011
*Azotobacter chroococcum* *Azospirillum brasilense* *Glomus fasciculatum*	Open field	Jensen's medium and N-free maltase medium	Soil (pH 7.12, organic carbon 9.6 g/kg)	Viable counts of microbial population in the rhizosphere increased significantly in all the treatments over control but decreased under chemical fertilizers treatment	Singh et al., 2013

Indirect viable counts on solid medium also allowed the assessment of the survival of endophytic trans-conjugant *Pseudomonas* sp. strains tagged with green fluorescent protein (GFP) in different tissues of poplar trees for 10 weeks (Germaine et al., 2004; **Table 1**). Since the plants were grown in a sterilized substrate but were not maintained under sterile conditions throughout the experiment, a number of indigenous endophytic strains were also isolated on growth medium. Therefore, to exclusively count the inoculated strains, only the colonies expressing *gfp* were enumerated by examining the plates under an epifluorescence microscope (Germaine et al., 2004).

Similarly, Kandel et al. (2015) used trans-conjugant GFP-tagged strains of *Burkholderia* sp., *Rhizobium tropici* PTD1, and *Rahnella* sp. WP5 to evaluate their colonization abilities in rice plants (**Table 1**). At 20 days after inoculation, the use of a selective growth medium allowed them to enumerate the total number of inoculated endophytes in the plant tissues. However, the use of axenic experimental conditions ensures ease of study and that only inoculated strains will be recovered.

### Greenhouse

Greenhouse experimental conditions could be considered a variation of farming in a controlled environment, which provides favorable growing conditions and protects crops from unfavorable weather and various pests. Therefore, this approach could be suitable for evaluating the viability of inoculated microorganisms by culture-dependent methods.

In pot greenhouse conditions, Wu et al. (2005) counted viable bacteria to demonstrate the successful colonization and the synergistic effect of beneficial rhizobacteria such as *Azotobacter* (*A.*) *chroococcum* and *Bacillus* (*B.*) (*B*. *megaterium* and *B*. *mucilaginous*) combined with mycorrhizal fungi belonging to the genus *Glomus* (*G*.) (*G. mosseae* or *G. intraradices*) in the rhizosphere of *Zea mays* plants (**Table 1**). The use of differential culture media allowed the detection and enumeration of groups of bacteria similar to the inoculants on the basis of their specific plant growth promoting activities, such as nitrogen fixation, phosphate and potassium solubilization.

Similarly, culture-dependent methods, based on the use of differentiation media for plant growth-promoting properties, were also useful to assess the persistence of bacterial (*A. chroococcum*, *B. megaterium* and *B. mucilaginous*) and fungal (*G. mosseae* or *G. fasciculatum*) consortia (Khalid et al., 2017; **Table 1**). The use of this approach demonstrated that the microbial concentration and root colonization of *Spinacia oleracea* L. was improved by the application of a consortium of microorganisms, suggesting the synergistic behavior of the strains.

The plate count method was also used to analyze the survival of five *Azotobacter* strains (ST3, ST6, ST9, ST17, and ST24) at different stages of wheat (*Triticum aestivum* L.) plant growth. These strains were inoculated in earthen pots containing saline soil under greenhouse conditions. The results of rhizosphere soil monitoring showed that the concentration of the inoculated strains increased up to 60 days of sampling (Chaudhary et al., 2013; **Table 1**). However, this approach did not allow the identification of microorganisms present in the culture at genus and species level in nonsterile condition. In fact, it is difficult to distinguish bioinoculants from indigenous microbial populations living in soils based on morphological characteristics.

Van Oosten et al. (2018) used viable microbial counts to assess the persistence of the inoculated *A. chroococcum* 76A in the rhizosphere of tomato plants cultivated under abiotic stress conditions (**Table 1**). A differentiating culture nitrogen-free medium for N fixers allowed them to demonstrate that the strain *A. chroococcum* 76A, inoculated at a concentration of approximately 10^6^ CFU/g, was able to grow in all experimental conditions, increasing by approximately one order of magnitude at the end of the experiment.

Interestingly, Solanki and Garg (2014) described a novel technique to enumerate viable cells of *A. chroococcum* in the unsterilized rhizoplane of *Brassica campestris* using a trans-conjugant strain of *A. chroococcum* Mac 27 containing a lacZ fusion (*A. chroococcum* Mac 27 L; **Table 1**). Using this approach, it was possible to monitor the growth and survival of the LacZ-tagged bacteria that formed blue-colored colonies on Burks medium containing X-gal.

### Field

Although the field represents the natural and real condition for assessing the effectiveness of a microbial consortium or biofertilizer in soil, it is difficult to differentially enumerate inoculated microorganisms in this experimental state by culture-dependent methods. However, some works have reported general results on the variation of microbial concentration in the rhizosphere of plants grown in agricultural fields.

Sharma et al. (2011) used a culture-dependent approach to assess microbial changes due to the application of a consortium formed by *A. chroococcum* AZ1 and AZ2 in association with *G*. *fasciculatum* and *G. mosseae* on apple plants grown in rainfed fields. As a general result, an increase in the concentration of bacteria and/or fungal strains in the inoculated tests was observed, although the results were more or less significant depending on the inoculant (used alone or in combination) and experimental conditions (**Table 1**).

A field experiment was also conducted to evaluate the inoculation effect of *Azotobacter*, *Azospirillum* (*Az.*), and AMF, either alone or in combination, on seedlings of apple cultivars. The viable counts of *A. chroococcum* and *Az. brasilense* in the rhizosphere were significantly higher in all the treatments than in the controls. In fact, the microbial concentration in the treatment with multi-inoculation of all the strains was significantly higher than those in all the other biological treatments but lower than that of the chemical fertilizer treatment (Singh et al., 2013; **Table 1**).

Culture-dependent methods have several advantages such as they are practical and useful techniques to quantify bioinoculants especially in sterile experimental conditions, and they allow to detect only viable cells and therefore bacterial inoculants that are competitive and able to persist overtime. Moreover, as reported in several works (Pitkäranta et al., 2007; Al-Awadhi et al., 2013; Ngom and Liu, 2014), it is difficult to detect the inoculated strain in unsterilized conditions. Culture-dependent methods cannot provide a comprehensive analysis of the endophytic ability of selected strains in unsterilized conditions since a portion of epiphytes that are resistant to sterilizing agents could determine an overestimation of their counts (Kandel et al., 2017). To explain the behavior of the bioinoculants in the natural soil ecosystem, culture-based methods should always be complemented with culture-independent approaches to examine the variations in the microbial community after inoculation treatment and to track the inoculated microbial strains.

## Microscopy-Based Techniques

Today, a wide range of microscopy-based techniques are available and have been used to detect microorganisms inoculated on plant tissues and to evaluate the colonization patterns of bacterial endophytes through molecular interactions and dynamics within living cells in specific vegetative tissues (Kandel et al., 2017).

Root colonization by bacteria and AMF has been studied by several types of microscopy, which can be divided into three major groups: light microscopy, electron microscopy and fluorescence microscopy.

### Optical Microscopy

Light microscopy is the most common microscopic technique for assessing microorganisms in root systems due to its low costs of purchasing, maintaining, and servicing (Hulse, 2018).

Bright-field light microscopy was employed by White et al. (2014), who developed a combination of stains to evaluate the bacterial colonization of seedling root tissues. This approach was based on the use of 3,3'-diaminobenzidine tetrachloride (DAB) to stain hydrogen peroxide associated with bacterial invasion of eukaryotic cells followed by counterstaining with aniline blue/lactophenol to stain protein in bacterial cells. This elementary technique allowed the visualization of bacteria and their eventual lysis in seedling roots, providing information on the defensive response of host cells and the bacterial degradation process (White et al., 2014).

Microscopy techniques that use different dyes are also usually used to assess mycorrhizal relationships with host plants. A wide number of staining procedures, which each have advantages and disadvantages, have been developed for studying AMF colonization, as extensively reported by Hulse (2018). Among these is a very simple, nontoxic, reliable and inexpensive staining technique for AMF colonization in root tissues; this technique is based on the use of an ink-vinegar solution after adequate clearing with KOH (Vierheilig et al., 1998). This solution stains all fungal structures, rendering them clearly visible by bright-field light microscopy.

The level of root colonization by mycorrhizal strains is usually evaluated using the microscopic procedure described by Phillips and Hayman (1970) and by Giovannetti and Mosse (Newman's intersection method, 1980). This method requires a stereomicroscope for observation; randomly dispersed roots are stained, placed on a grid in a 9-cm Petri plate and quantified by counting the number of intersections between grid lines and colonized roots. Although this method is strongly influenced by operator skill, it could provide sufficient information to evaluate the mycorrhizal colonization level. In fact, the gridline intersect method has been extensively used in many works to assess and quantify root colonization of mycorrhizal fungi (Sharma et al., 2009; Sharma et al., 2011; Sharma et al., 2012; Singh et al., 2013).

### Electron Microscopy

Electron microscopy was further developed into scanning electron microscopy (SEM), which can be used to examine plant surfaces and microorganisms at high resolution, highlighting the adhesion of microbial cells to plant tissues. SEM was used to observe chickpea root colonization by *A. chroococcum* and *Trichoderma viride* (Velmourougane et al., 2017; **Table 2**). The plants were cultivated in sterile media composed of sand and vermiculite (1:1), and samples were taken at 40 days post inoculation. SEM microphotographs revealed the proliferation of *Azotobacter* cells, both individually and attached to the fungal mycelia. SEM observations have also highlighted the production of exopolysaccharides by *A. chroococcum.* These polymers improve the survival of EPS-producing microbial cells in natural ecosystems, exhibit beneficial effects in plant growth promotion and abiotic stress (Gauri et al., 2012; Van Oosten et al., 2017) and could be interesting for biopolymer production (Ventorino et al., 2019). Although SEM produces 3D images, it provides information only on surface morphology and colonization and is not as powerful as transmission electron microscopy (TEM). Although TEM is not considered a user-friendly technique since sample preparation is complex and time consuming, it is the most powerful microscopy technique, with a maximum potential magnification of 1 nanometer. TEM allows 2D ultrahigh resolution images to be obtained, providing information about the internal structure of a root sample; therefore, it is useful to establish endophytic interaction as reported by Singh and Sharma (2016). Hairy roots of *Arnebia hispidissima* were inoculated *in vitro* with five different *A. chroococcum* strains (**Table 2**). After 10 days of incubation, TEM showed that *A. chroococcum* strains were only inside hairy roots of inoculated plants, revealing the endophytic ability of *A. chroococcum* strains. However, since TEM allows only a small area of a sample to be explored, which provides information about the inner part of a sample, and SEM can explore a larger external area, these two techniques could be used in combination to obtain better detailed results about the rhizosphere environment and inoculant colonization (Thokchom et al., 2017).

**Table 2:** Microscopy-based techniques used to monitor plant growth-promoting bacteria and root interaction.

Strains	Experimental conditions	Methods	Plant substrate		Results	Reference	
*Burkholderia gladioli*	Laboratory experiment on *Panicum virgatum*	Bright field microspy	Water agar plates		Bacterial cells adhered to surfaces of root hairs and root epidermal parenchyma	White et al., 2014	
*Azotobacter chroococcum* W5 *Trichoderma viride* ITCC 2211	Pot (day/night temperature 22–24/18°C, humidity 60%)	SEM	Sterile sand and vermiculite (1:1)		Presence of *Azotobacter* cells, both individually both attached to the fungal mycelia, on root tissues	Velmourougane et al., 2017	
*Azotobacter chroococcum* ATCC9043 *Azotobacter chroococcum* BCRC10599 *Azotobacter chroococcum* CCRC10599 *Azotobacter chroococcum* DSM2286 *Azotobacter chroococcum* IAM12666	*In vitro* assay on *Arnebia hispidissima* (25 ± 1°C, 60% relative humidity, 10 days)	TEM	MS culture medium		Endophytic interaction between bacterial strains and hairy roots	Singh and Sharma, 2016	
*Azospirillum* spp. *Azoarcus* spp. *Azorhizobium* spp.	Controlled conditions (22°C; 16-h/8-h light/dark; relative humidity 75%)	ESEM	MS agar medium		Colonization of root cavities, bacterial biofilm formation, colonization of inner root tissues	Dal Cortivo et al., 2017	
*Azotobacter chroococcum* Mac 27L	Phytotron chamber (12 h light, ca. 30,000 lux, 15–17°C/8–10°C day/night temperature, 28 days)	Immuno-fluorescence microscopy		Semisolid nutrient media	Bacteria were clearly detectable after 7 days of inoculation	Narula et al., 2007	
*Burkholderia* sp. WPB *Rhizobium tropici* PTD1 *Rahnella* sp. WP5	Axenic conditions in growth chamber	GFP		N-free MS agar	Bacterial cells reside outside plant tissues in the apoplastic spaces and xylem tissue of rice plants	Kandel et al., 2015	
*Pseudomonas* sp. VM1449 *Pseudomonas* sp. VM1450 *Pseudomonas* sp. VM1453	Pots (20–25°C, 16-h light/8-h dark)	GFP		Sterile compost/vermiculite substrate (3:1 ratio)	GFP-tagged cells were clearly visible in the rhizosphere and on different root tissues	Germaine et al., 2004	
*Pseudomonas fluorescens* SBW25	Laboratory experiment on 5 days growth lettuce	GFP		Transparent soil of particles of Nafion (polymer with a low refractive index)	Colonization of root surfaces, rhizoplane, and surfaces of Nafion particles	Downie et al., 2014	
*Azotobacter chroococcum* Avi2	*In vitro* assay on sterile rice seedlings (14-h light cycle, 30 ± 2°C, 7 days)	FRET-based technique		MS agar medium	Intracellular roots colonization (green fluorescence emitted by bacterial cells and blue fluorescence emitted by root tissues)	Banik et al., 2016	
*Azotobacter chroococcum* 67B *Azotobacter chroococcum* 76A	*In vitro* assay (sterile conditions)	Fluorescent Al^3+^-siderophore complex combined with CLSM		Pots containing a growth medium added of 2 mM of Al^3+^	Ability of the two bacterial strains to colonize tomato roots	Viscardi et al., 2016
*Sphingomonas azotifigens* DSMZ18530	Gnotobiotic conditions in controlled-environment chamber (16-h light/8-h dark, 18–23°C)	GFP		Modified Evans medium supplemented with 8% agar	Visualization and localization of bacterial strain in different parts of annual ryegrass plants (preferentially localized along root hairs and in stem epidermis)	Castanheira et al., 2017
*Pseudomonas* sp. G1Dc10 *Paenibacillus* sp. G3Ac9	Gnotobiotic conditions in controlled-environment chamber (16-h light/8-h dark, 18–23°C)	FISH/Confocal laser-scanning microscopy		Modified Evans medium supplemented with 8% agar	Visualization and localization of bacterial strains in different parts of annual ryegrass plants (preferentially localized along root hairs and in stem epidermis)	Castanheira et al., 2017

FRET, fluorescence resonance energy transfer; SEM, scanning electron microscopy; TEM, transmission electron microscopy; ESEM, environmental scanning electron microscopy; GFP, green fluorescent protein; FISH, fluorescence in situ hybridization.

Environmental scanning electron microscopy (ESEM) is another powerful method to evaluate the survival of a bacterial inoculant and its ability to colonize plant tissues. It provides new possibilities compared to conventional SEM and enables the investigation of nonconductive and hydrated samples without complex histological preparation steps (i.e., air drying, chemical fixation, dehydration, and coating), which are critical in conventional SEM (Stabentheiner et al., 2010). This approach was recently used by Dal Cortivo et al. (2017) to evaluate the colonization level of a commercial biofertilizer containing a bacterial consortium on wheat in sterile conditions (**Table 2**). ESEM imaging revealed good survival rates as well as external and internal colonization of leaf and root tissues by a bacterial consortium.

Although electron microscopy allows clear visualization of cells outside and inside plant tissues at a very high resolution, this technique can be used only in limited sterile conditions since it is unable to distinguish bioinoculants from indigenous microbial populations living in soils.

### Fluorescence Microscopy

Fluorescence microscopy has become an essential technique in biology for the study of living tissues or cells. Although this method requires more complex and expensive instrumentation than conventional transmitted-light microscopy, it is widely used for the detection of bacteria inside plant tissues. This is possible because fluorescence microscopy reveals the position of fluorescent substances that were previously introduced into living cells. Several fluorescent dyes and protein tags and other methods to fluorescently label cells can be employed, providing a range of tools to track a microbial inoculant.

Narula and coworkers (2007) proposed the use of serological methods such as double-antibody sandwich enzyme-linked immunosorbent assay and immunofluorescence as potential techniques for investigating the colonization behavior of bioinoculants. They revealed the presence of *A. chroococcum* Mac 27 L in root fragments of hydroponically grown wheat plants using immunofluorescence (**Table 2**). However, one of the most commonly used methods for tracking endophytic inoculated bacteria within plant tissues is the use of GFP, which emits fluorescent green light when irradiated with blue light or near-ultraviolet (UV) light (Wang et al., 2015). The detection and quantification of GFP-tagged strains is possible using epifluorescence microscopy (Leff and Leff, 1996), confocal laser-scanning microscopy (CLSM) (Götz et al., 2006; Fan et al., 2011; Krzyzanowska et al., 2012), flow cytometry (Elvang et al., 2001), and UV exposure for solid agar plates (Errampalli et al., 1999). The use of GFP allowed the evaluation the colonization abilities of tagged *Burkholderia* sp., *Rhizobium tropici* PTD1, and *Rahnella* sp. WP5 in rice plants grown in N-free MS agar for twenty days in a growth chamber (Kandel et al., 2015; **Table 2**). The presence of three inoculated GFP-tagged endophytic *Pseudomonas* sp. strains in different poplar tree tissues (leaf, stem and root) was verified by Germaine et al. (2004) using an epifluorescence microscope (**Table 2**). An innovative transparent soil made of a polymer with a low refractive index was used by Downie et al. (2014) to evaluate the abundance of GFP-tagged *P. fluorescens* SBW25 on *Lactuca sativa* roots (**Table 2**). The transparency of the substrate allowed them to capture images using confocal microscopy, which showed a high bacterial abundance on the root tips and at root branching zones. Although the use of GFP-tagged microbial strains has various advantages, such as no influence of autochthonous bacteria and the possibility of *in situ* detection, it can be used only in laboratory/greenhouse experiments since this method requires that the microbe be transformed before any application (Compant and Mathieu, 2013). In addition, the visualization of GFP expression is sometimes difficult due to the autofluorescence of the plant cell walls (Germaine et al., 2004), and it is difficult to detect inoculated microbes *in situ* because of interference by soil particles (Quadt-Hallmann and Kloepper, 1996). Finally, the procedure for the transformation of the GFP-plasmid involves exposure to CaCl_2_, which promotes cyst formation in some endophytic strains, such as *A. chroococcum*; therefore, the procedure is unsuccessful in certain organisms. This is the main reason for developing an alternative procedure based on fluorescence resonance energy transfer to visualize endophytes inside plant tissues when the use of GFP is restricted. This technique is based on the use of a novel specific rhodamine-pyrene conjugate as an Al^3+^ selective colorimetric and fluorescence sensor to visualize the endophytes with minimum interference of background autofluorescence, unlike GFP tagging. The fluorescence resonance energy transfer-based technique was used by Banik et al. (2016) to track the *A. chroococcum* Avi2 strain after inoculation on sterile rice seedlings (**Table 2**). The results showed intracellular root colonization by the *A. chroococcum* Avi2 strain since a clear and stable green fluorescence was emitted by bacterial cells and detected by fluorescence microscopy, whereas a blue fluorescence was emitted by root tissues, proving the feasibility of this approach. In fact, the authors demonstrated that the rhodamine–pyrene conjugate was an excellent fluorescence ligand that was green-shifted only by the Al^3+^-treated bacterial cells since it was able to detect only intercellular Al^3+^ (Banik et al., 2016).

The fluorescent Al^3+^-siderophore complex produced by *A. chroococcum* strains was used by Viscardi et al. (2016) in combination with CLSM to assess the rhizocompetence of inoculated bacteria on tomato plants under sterile conditions *in vitro*, demonstrating the ability of the two selected bacteria to colonize plant roots (**Table 2**).

To determine the colonization ability of microbes on and inside plants, other methods, such as fluorescence *in situ* hybridization (FISH), have been employed. FISH is a molecular method based on the use of fluorescently tagged oligonucleotide probes, which are able to bind ribosomal RNA sequences to target metabolically active and intact cells (Moter and Gobel, 2000), combined with microscopy techniques such as epifluorescence microscopy (Compant and Mathieu, 2013) or CLSM (Rothballer et al., 2003; Wu et al., 2008). The range of available and developed probes for the detection of microbial cells using universal probes or strain-specific probes limits this technique. In addition, the long and complex sample preparation protocol (Moter and Gobel, 2000) could represent a disadvantage of this approach. Recently, the colonization ability of a multistrain inoculant composed of *Pseudomonas* sp. G1Dc10, *Paenibacillus* sp. G3Ac9 and *S. azotifigens* DSMZ 18530 on annual ryegrass plants was analyzed using FISH combined with CLSM (Castanheira et al., 2017; **Table 2**). However, in plant tissues, FISH showed several limitations due to weak and/or unsuccessful hybridization signals of the probe. In fact, it was reported that in the FISH method, a low signal intensity of some of the detected microbes can occur due to a low cellular concentration of the target molecules or due to the low *in situ* accessibility of rRNA regions for singly labeled probes, thus preventing their successful visualization in plants (Wagner et al., 2003; Compant and Mathieu, 2013). Therefore, to overcome this problem, a combination of FISH, GFP-labeling methods and CLSM was employed. In detail, the use of FISH to detect a GFP-labeled *S. azotifigens* strain increased the signal, improving the visualization of bacterial cells and enabling the visualization and localization of inoculated strains in different parts of plants (Castanheira et al., 2017).

Although bioinoculants inside plant tissues can be clearly visualized by microscopy-based techniques, these techniques can suffer from several limitations (Pantanella et al., 2013; Emerson et al., 2017). For example, it is not always possible to distinguish living cells from dead cells by direct observation, and the autofluorescence of the plant cells sometimes makes it difficult to visualize microbial cells inside different plant tissues. Moreover, tagged microbial cells should be used only in limited and controlled experimental conditions (growth chamber and greenhouse) since it is not always permitted the dispersion of modified microorganisms in the environment, preventing the evaluation of survival and colonization ability of the bioinoculant in natural real ecosystems.

## Molecular Approaches

Methods based on the analysis of nucleic acids extracted directly from soil/rhizosphere samples have been developed to overcome cultivation limitations. In fact, the development of molecular tools allows new species of unculturable microorganisms associated with the root system to be discovered or helps to understand the ecological function of several microbial species (Lebeis et al., 2012; Bulgarelli et al., 2013). The total genetic material recovered directly from soil samples represents the soil metagenome (Daniel, 2005), and metagenomics is the field of molecular genetics and ecology that studies this “collective” genome to determine the phylogenetic and functional gene complements of a sample (Pershina et al., 2013; Jansson, 2015). The development of metagenomic techniques, including the use of DNA probes (Bouvier and del Giorgio, 2003), polymerase chain reaction (PCR)-based techniques (Ruppel et al., 2006) and next-generation sequencing (NGS, Mardis, 2008), has greatly increased the ability to track microorganisms in natural environments (Ahmad et al., 2011). However, considering the high microbial diversity and the complex environmental matrix, DNA extraction is a fundamental step that could affect the detection and quantification of microbial taxa inferred from metagenomic sequences in all molecular methods; therefore, specific microbial groups can be underrepresented (Morgan et al., 2010; Montella et al., 2017). Currently, two main approaches are used for microbial DNA extraction from soil (Lombard et al., 2011): i) direct extraction, based on the direct lysis of microbial cells inside the soil matrix followed by DNA extraction and purification; and ii) indirect extraction, based on the initial recovery of microbial cells from the soil samples followed by lysis and DNA extraction and purification. Although both DNA extraction approaches are suitable for metagenomic analysis, they have different advantages and drawbacks in terms of DNA quantity and quality, even when starting from the same matrix (Ventorino et al., 2015; Montella et al., 2017), as extensively reported by Lombard et al. (2011), depending on the soil type. Therefore, when beginning a metagenomic analysis of soil, it is critical to define which DNA extraction method will be optimal by considering the subsequent genomic analysis (Lombard et al., 2011). For a more detailed discussion on this topic see Lombard et al. (2011).

### PCR-Based Methods

In recent decades, several molecular approaches, such as quantitative real-time PCR (qPCR), denaturing gradient gel electrophoresis (DGGE), automatic ribosomal interspace spacer analysis, amplified ribosomal DNA restriction analysis, and NGS, have been used to investigate the presence of microbial inoculant in the soil system and to determine its impact on the rhizosphere community (Ciccillo et al., 2002; Steddom et al., 2002; Gamalero et al., 2003). These approaches allow the detection of specific microorganisms and/or the abundance of different microbial populations or species on the basis of the amplification of specific genes. Among these techniques, qPCR is a sensitive and suitable approach for determining the abundance of functional genes from soil-derived DNA and RNA (Fiorentino et al., 2016), and it has therefore been extensively used to track and quantify inoculated strains in soil systems (Providenti et al., 2009; Timmusk et al., 2009). For instance, Sorte et al. (2014) used this method to design specific PCR primers targeting a 16S rRNA variable region to specifically measure the abundance of *Gluconacetobacter diazotrophicus* following coinoculation with other diazotrophic strains in sugarcane plants grown under field conditions (**Table 3**). The validation of employed species-specific primers allow the use of this method to evaluate the occurrence of endophytic diazotrophic *G. diazotrophicus* species in any soil type and plant tissue. A qPCR protocol was also developed by Couillerot et al. (2010) for the strain-specific quantification of *Az. brasilense* UAP-154 and CFN-535 in the maize rhizosphere using BOX-based sequence characterized amplified region markers, although the detection limit ranged from 10^4^ to 10^8^ CFU g^-1^ (**Table 3**). The success of this approach has led other authors to use it. In fact, strain-specific primers recovered from draft genome sequence analysis were employed for qPCR to quantify *Az. brasilense* FP2 in wheat roots as well as to assess its competitiveness following coinoculation with other PGPR (Stets et al., 2015; **Table 3**). All of these works demonstrate the high effectiveness and specificity of this culture-independent approach based on the use of strain-specific primers, allowing rapid and inexpensive detection of bioinoculants in the plant rhizosphere for monitoring and quantification purposes, which is also useful in nonsterile and uncontrolled conditions.

**Table 3:** Molecular approaches used to monitor plant growth‒promoting bacteria and root interaction.

Strains	Experimental conditions	Method	Plant substrate	Results	Reference
*Gluconacetobacter diazotrophicus*	Field experiment on sugarcane	qPCR	Soil (pH 5.3, P 6.1, 6.8 mg/dm^3^, K 44 mg/dm^3^, organic matter 1.3%)	Quantification of bacterial cells in plant tissues using species-specific primers	Sorte et al., 2014
*Azospirillum brasilense* UAP-154 *Azospirillum brasilense* CFN-535	Pots in greenhouse on maize (18-h/6-h light/dark, 18–22°C, 10 days)	qPCR	Sieved non sterile soil from La Côte St André adjusted to 20% (w/w) water content	Quantification of bacterial cells in the rhizosphere using primers designed on strain-specific SCAR markers	Couillerot et al., 2010
*Azospirillum brasilense* FP2	Wheat plants germinated under sterile conditions, incubated in a greenhouse (14-h light/10-h dark, 23°C, humidity above 50%)	qPCR	Hoagland solution and quartz beads in glass tubes	Quantification of *A. brasilense* FP2 in the rhizosphere under sterile conditions	Stets et al., 2015
*Azospirillum brasilense* FP2 alone or co-inoculated with *Azospirillum brasilense* NH, *Herbaspirillum seropedicae* Z67, *Gluconacetobacter diazotrophicus* DSM 5601, *Azospirillum lipoferum* DSM 1691	Wheat plants germinated under nonsterile conditions, incubated in a greenhouse (14-h light/10-h dark, 23°C, humidity above 50%)	qPCR	Quartz beads in glass tubes	Quantification of *A. brasilense* FP2 in the rhizosphere even under nonsterile conditions and when coinoculated with other rhizobacteria using strain-specific primers	Stets et al., 2015
*Burkholderia* sp. J62 *Pseudomonas thivervalensis* Y-1-3-9	Pot with rape plants (30.4 ± 4.6°C/18.3 ± 3.2°C day/night, relative humidity 67.5 ± 12.9%)	PCR-DGGE	Contaminated soils (0.50 mg/kg of Cd and 100 mg/kg of CdSO_4_)	Inoculated bacteria were detected in the root interiors and rhizosphere soils	Chen et al., 2013
*Azospirillum brasilense* Cd	Shade house with sorghum (temperature ~29°C, light intensity of ~1,000 μmol photons m^2^/s, 20 days; three crop cycles)	PCR-DGGE	Highly degraded alluvial desert soil	Persistence of the inoculant within the bacterial community of the rhizosphere of sorghum plants by purification and sequencing od DGGE bands	Lopez et al., 2013
*Dyadobacter* sp.	Pot trial in a net house (sampling at 30, 45, 60, and 90 days)	PCR-DGGE - qPCR	Soil (pH 7.5, oxidazable organic carbon 0.3–0.5%; phosphorus pentoxide <22 kg/ha, ammonia 15 kg/ha, nitrate 4 kg/ha)	Quantification of diazotrophic abundance by qPCR and persistence of inoculant in the soil by detection of a specific DGGE band.	Kumar et al., 2018
*Streptomyces* sp. AH-B	Containers with dry natural soil sprayed with quinclorac solution	NGS	-	*Streptomyces* sp. AH-B became the dominant species following inoculation in quinclorac-contaminated soil	Lang et al., 2018
*Bacillus amyloliquefaciens* FZB42	Field trial on lettuce rhizosphere	WGS - metagenomic study	Soil (alluvial loam, total N 112 mg/100 g, P 32.3 mg/100 g, K 17.4 mg/100 g, Mg 9.1 mg/100 g, pH 6.5	Presence of the strain in the rhizosphere over 5 weeks in field. Marginal changes in the bacterial community after inoculant application.	Kröber et al., 2014

SCAR, sequence characterized amplified region; qPCR, quantitative real-time PCR; DGGE, denaturing gradient gel electrophoresis; NGS, next-generation sequencing; WGS, whole-genome sequencing.

The addition of bioinoculants in a soil could determine variations in the native microbial community structure, as recently reported by Fiorentino et al. (2018). PCR-DGGE followed by sequence analysis of bands is a metagenomic approach able to describe changes in soil microbial communities after inoculation of bacterial or fungal strains as well as to test the persistence of microbial inoculant in the soil. By DGGE and gene sequence analyses, Chen et al. (2013) detected heavy metal-resistant *Burkholderia* sp. J62 and *P*. *thivervalensis* Y-1-3-9 in both root interiors and rhizosphere soil of *Brassica napus* L., demonstrating their influence on the rape-associated bacterial community structures in artificially Cd-contaminated soil (**Table 3**). The presence of *Az. brasilense* Cd (DSM 1843) in the rhizosphere of sorghum plants was monitored by Lopez et al. (2013) by gene sequencing of DGGE bands for three crop cycles (**Table 3**), highlighting its rhizocompetence against indigenous populations. However, since DGGE allows us to distinguish microbial populations at the species level, when the experiments are carried out in nonsterile soil, it is difficult to ensure that a sequence of bands originated from inoculated microbial strains or from other autochthonous strains belonging to the same species. Therefore, DGGE analysis is usually performed in combination with other techniques, such as FISH (Lopez et al., 2013), GFP (Piromyou et al., 2013), SEM, and TEM (Thokchom et al., 2017). In some cases, the combination of DGGE and qPCR is a suitable approach to investigate the abundance of specific microbial groups and the survival of bioinoculants in the soil, as recently reported by Kumar et al. (2018) in a pot trial-based study (**Table 3**). In this case, DGGE was a useful approach to check bioinoculants because no band corresponding to inoculated *Dyadobacter* sp. was recovered in the control soil.

### Next-Generation Sequencing

In recent decades, the development of massive DNA sequencing technology, known as NGS, and bioinformatic tools has provided a powerful alternative to other molecular studies of microbial ecology in natural environments, enabling the study of taxonomic diversity at a high resolution (Ventorino et al., 2018). Indeed, analyzing the rhizosphere microbiome with the high-throughput sequencing approach has different prospective results that could allow understanding the community structure of root-associated bacteria and, as a consequence, novel bacteria with plant growth promoting traits to be discovered. This approach could also help to understand changes in the microbial community dynamics and structure after inoculation treatments. NGS could be performed following two different approaches: i) amplicon sequencing based on the amplification of phylogenetic marker genes, usually hypervariable regions from small-subunit ribosomal RNA genes (i.e., 16S rRNA), followed by bioinformatic analysis; ii) shotgun sequencing based on random sequencing across entire genomes followed by genome assembly and bioinformatics analysis. The construction of environment-based libraries was a major advance in soil metagenomics, and these libraries could be screened by functional and sequence-based approaches to clarify several functions of organisms in soil communities and to simplify genomic analyses of uncultured soil microorganisms (Garza and Dutilh, 2015). Recently, NGS of 16S rRNA genes was used to evaluate the behavior of the strain *Streptomyces* sp. AH-B after it was inoculated in quinclorac-contaminated soil, as well as its influence on soil microbial communities (Lang et al., 2018). After alignment, sequences were clustered into operational taxonomic units (OTUs) at 97% identity, which revealed that *Streptomyces* sp. AH-B became the dominant species following inoculation and that the bacterial and fungal diversity in treated soil was higher than that in the control, probably due to the degradation activity of inoculant that could reduce quinclorac toxicity to microorganisms. However, due to the high and complex biodiversity of soil microbial communities and the presence of various PCR and library preparation inhibitors, such as humic substances, full coverage of the soil metagenome is a difficult task. Moreover, the identification of OTUs at 97% identity thresholds allow to discriminate microbial populations at the species level but not at the strain level, so different strains with different plant growth promoting activities could be pooled together. In addition, identical OTUs do not necessarily mean the same species, since there are several databases for microbial identification, and it could be difficult to compare different studies, since the determination of sequences depends on sequences entered into DNA collections. Finally, high-quality DNA extraction for NGS is challenging for soil studies and is dependent on the extraction method and soil characteristics (Daniel, 2005).

### Whole-Genome Sequencing and Pangenome

The determination of the entire genomic DNA sequence at a single time sequence [whole-genome sequencing (WGS)] of a microbial strain could be a powerful approach to investigate the potential PGP activities of a strain as well as its plant colonization and survival efficiency in the rhizosphere, leading to the identification of specific genes related and involved in plant–microbe interactions. In recent years, this approach was used to characterize new PGPR strains. Functional annotation of WGS of the strain *B. aryabhattai* AB211 revealed the presence of common genes involved in PGP activities and in abiotic/biotic stress tolerance as well as genes conferring resistance to oxidative stresses in plants demonstrating its high potential as a PGPM (Bhattacharya et al., 2017). However, the presence of PGP-related genes is essential but not sufficient for a bacterium to exert beneficial effects on plant growth in a real environment. In fact, although the presence of key attributes essential for possible colonization and interaction with the host plant were recovered in two *Rhodopseudomonas palustris* strains (PS3 and YSC3), these strains exhibited different expression patterns of genes related to PGP activities, probably due to the different physiological responses of these strains to specific compounds in the root exudates that act as signal molecules (Lo et al., 2018). Therefore, the effectiveness of PGP activities of a specific strain could also be affected by the different exudates released into the soil by different plants.

WGS could also be used in combination with metagenomic studies to identify microbial strains in the soil metagenome. Using this approach, the presence of the plant-associated strain *B. amyloliquefaciens* FZB42 on lettuce was assessed by Kröber et al. (2014; **Table 3**). Fragment recruitments of metagenome sequence reads on the referenced genome sequence of *B. amyloliquefaciens* FZB42 following shotgun sequencing of whole rhizosphere microbial communities of inoculated plants evidenced that the strain was present for over 5 weeks. Therefore, the combination of WGS and shotgun sequencing could be a suitable approach to identify the persistence of a microbial inoculant in the rhizosphere of plants grown in a natural environment.

Another method for the detection and identification of key genes responsible for the adaptation and evolution of a microbe as an endophyte is the pangenome. The pangenome can be defined as the entire genetic repertoire of a species; it comprises a core genome, which is composed of the genes present in all strains of the species, and an accessory genome, comprising the genes that are unique to specific strains (Mira et al., 2010; De Maayer et al., 2014). By analyzing the pangenome of eight sequenced *Pantoea ananatis* strains isolated from different sources, De Maayer and coworkers (2014) identified proteins with a potential role in plant–microbe interactions. Despite the large amount of information that could be retrieved from the pangenome, this method is still rarely used for studying the genetic traits of endophytes since it is based on the cultivation of microbial strains; therefore, nonculturable endophytes remain unexplored (Kaul et al., 2016).

Recently, Albanese and Donati (2017) proposed a novel method (StrainEst) based on the use of single-nucleotide variant profiles of the referenced available genomes of selected species to identify and quantify the strains of interest present in metagenomic samples. This novel approach could be useful to highlight differences at the strain level that could allow us to track a microbial inoculant in the rhizosphere.

The increasing database of sequenced microbial genomes also allows genome-wide computational searches for clustered, regularly interspaced short palindromic repeats (CRISPRs) in microbial species (Sorek et al., 2008). These repetitive sequences have been detected in a wide number of bacterial and archaeal genomes (Horvath and Barrangou, 2010), including PGPM. CRISPRs are usually used as molecular markers for the detection of pathogenic microbes or for the evaluation of phage-resistance mechanisms in bacteria (Sorek et al., 2008). Although the CRISPR approach has been applied to plant-soil environments only to detect plant pathogenic strains such as *Erwinia amylovora* (McGhee and Sundin, 2012), it could be exploited in the future for developing molecular markers to monitor PGPR for plant–microbe interactions (Rilling et al., 2019).

The development of molecular techniques based on the analysis of nucleic acids provides an approach useful to understand plant–soil–microbe interactions. These methods have greatly increased the ability to track microorganisms in natural environments and some of them allow a rapid and inexpensive detection of bioinoculants in the plant rhizosphere for monitoring and quantification purposes overcoming cultivation limitations. The use of one or a combination of these methods allow the investigation of the abundance of specific microbial groups and the survival of bioinoculants in the soil as well as variations in the native microbial community dynamics and structure (Kumar et al., 2018). Although DNA-based approaches have improved our knowledge of microbial ecology, they are not able to differentiate between live and dead cells. Therefore, it is recommended to use them in combination with conventional methods, such as culture enumerations, for investigating bacterial ecology in natural habitats. Finally, molecular methods are highly influenced by DNA quality and quantity that is dependent on the extraction method and soil characteristics (Daniel, 2005; Lombard et al., 2011).

## Conclusion

Assessing the root colonization of inoculants with beneficial effects on plant growth as well as their persistence over time in a soil is a critical issue in sustainable agriculture. Currently, several approaches that use culture-dependent, microscopic and molecular methods have been developed to follow bioinoculants in the soil and on the plant surface. However, to ensure good results in microbiological analysis, the first fundamental prerequisite is the correct soil sampling and sample preparation for the different methodological approaches that will be assayed.

Although plant colonization of bacterial endophytes can be assessed by microscopy-based techniques through molecular interactions and dynamics within living cells in a specific vegetable tissue, the measurement of the persistence of inoculants in soil poses technical difficulties, as the inoculant needs to be identified from a complex community. Methods to detect persistence include cultural enumeration or molecular approaches using PCR-based methods and next-generation sequencing. Culture-dependent methods are commonly used to estimate the persistence of inoculated bacteria in soil and/or rhizosphere, mainly for their ease of use, but this analysis is limited since it is difficult to represent the high diversity of bacteria on culture media and, at the same time, it is difficult to differentiate inoculated organisms from native populations based on morphological characteristics. Therefore, culture-dependent methods are especially useful when the experiment is carried out in sterile conditions to avoid interference by native microbial populations living in the soil. Molecular analysis allows the detection of bioinoculants or their activity in soil and contemporaneous evaluation of the effect of rhizosphere engineering on native microbial communities. However, most of the molecular techniques are based on the preliminary genomic characterization of the microbial strain used as inoculant and the specific molecular markers of the strain for its detection in the soil metagenome. Molecular approaches help to improve our knowledge of microbial ecology, but they cannot be considered as a substitute for more conventional methods, such as culture enumerations. In fact, if DNA is analyzed, there is the disadvantage of the inability to differentiate between live and dead cells; therefore, these methods should be considered complementary for investigating bacterial ecology in natural habitats. Future perspectives in the assessment of colonization and soil persistence should have a polyphasic approach combining several molecular and microbiological techniques to allow the tracking of inoculated strains or microbial consortia.

Moreover, a microscopy-based approach allows us to obtain a picture of bacterial colonization outside and inside plant tissues, but it is not possible to always distinguish living cells from dead cells by direct observation. The autofluorescence of the plant cells and interference by soil particles make it difficult to visualize microbial cells inside different plant tissues. Tagged microbial cells should be used only in limited and controlled experimental conditions (growth chamber and greenhouse), and the evaluation of the survival and colonization ability of an inoculant in a natural real ecosystem cannot be performed because the strains could be released into the environment.

All the described methods have advantages and disadvantages and provide only partial results, and most of them are time-consuming, expensive and unable to detect specific inoculated microbial strains. Therefore, to better explain the behavior of bioinoculants in the natural soil ecosystems, culture-dependent and culture-independent (molecular and microscopic approaches) methods should be used in combination to examine the variations in microbial communities after inoculation treatment and to track the inoculated microbial strains in different systems.

The main challenge for the application of PGPM as bioinoculants in unsterilized greenhouse or field conditions is the establishment of effective methods for the assessment of plant colonization and soil persistence. Moreover, modern soil microbiology lacks efficient methods for the detection and estimation of the effective PGP activities that inoculated strains have on the soil. This is another main bottleneck in the use of microbial inocula for rhizosphere engineering. Therefore, the development of specific and easy methodologies for the evaluation of PGP activities could help to understand what actually occurs in a natural soil system during plant–soil–microbe interactions.

## Author Contributions

IR and VV wrote the manuscript in collaboration. VV and OP conceived the concepts of the manuscript.

## Funding

This work was supported by FSE-FESR PON R&I 2014-2020, PhD program on “Sustainable agricultural and forestry systems and food security” - XXXIII cycle.

## Conflict of Interest

The authors declare that the research was conducted in the absence of any commercial or financial relationships that could be construed as a potential conflict of interest.

## References

[B1] Ahmad F. Husain F. M. Ahmad I. (2011). “Rhizosphere and root colonization by bacterial inoculants and their monitoring methods: a critical area in PGPR research,” in Microbes and Microbial technology. Eds. Ahmad I. Ahmad F. Pichtel J. (New York, NY: Springer), 363–391. 10.1007/978-1-4419-7931-5_14

[B2] Al-Awadhi H. Dashti N. Khanafer M. Al-Mailem D. Ali N. Radwan S. (2013). Bias problems in culture-independent analysis of environmental bacterial communities: a representative study on hydrocarbonoclastic bacteria. SpringerPlus 2, 369. 10.1186/2193-1801-2-369 24040582 PMC3769543

[B3] Albanese D. Donati C. (2017). Strain profiling and epidemiology of bacterial species from metagenomic sequencing. Nat. Commun. 8, 2260. 10.1038/s41467-017-02209-5 29273717 PMC5741664

[B4] Andreote D. F. João L. A. Welington L. A. (2009). Assessing the diversity of bacterial communities associated with plants. Braz. J. Microbiol. 40, 417–432. 10.1590/S1517-83822009000300001 24031382 PMC3768544

[B5] Banik A. Mukhopadhaya S. K. Sahana A. Das D. Dangar T. K. (2016). Fluorescence resonance energy transfer (FRET)-based technique for tracking of endophytic bacteria in rice roots. Biol. Fertil. Soils 52, 277–282. 10.1007/s00374-015-1064-6

[B6] Barillot C. D. C. Sarde C. O. Bert V. Tarnaud E. Cochet N. (2013). A standardized method for the sampling of rhizosphere and rhizoplan soil bacteria associated to a herbaceous root system. Ann. Microbiol. 63, 471–476. 10.1007/s13213-012-0491-y

[B7] Bhattacharya C. Bakshi U. Mallick I. Mukherji S. Bera B. Ghosh A. (2017). Genome-guided insights into the plant growth promotion capabilities of the physiologically versatile *Bacillus aryabhattai* strain AB211. Front. Microbiol. 8, 411. 10.3389/fmicb.2017.00411 28377746 PMC5359284

[B8] Bouvier T. del Giorgio P. A. (2003). Factors influencing the detection of bacterial cells using fluorescence *in situ* hybridization (FISH): a quantitative review of published reports. FEMS Microbiol. Ecol. 44, 3–15. 10.1016/S0168-6496(02)00461-0 19719646

[B9] Brandt E. Kluepfel D. (1991). The release and tracking of genetically engineered bacteria in the environment. Phytopathology 81, 348–352. 10.1016/0958-1669(95)80048-4

[B10] Bulgarelli D. Schlaeppi K. Spaepen S. Ver Loren van Themaat E. Schulze-Lefert P. (2013). Structure and functions of the bacterial microbiota of plants. Annu. Rev. Plant Biol. 64, 807–838. 10.1146/annurev-arplant-050312-120106 23373698

[B11] Castanheira N. L. Dourado A. C. Pais I. Semedo J. Scotti-Campos P. Borges N. (2017). Colonization and beneficial effects on annual ryegrass by mixed inoculation with plant growth promoting bacteria. Microbiol. Res. 198, 47–55. 10.1016/j.micres.2017.01.009 28285661

[B12] Chaudhary D. Narula N. Sindhu S. S. Behl R. K. (2013). Plant growth stimulation of wheat (*Triticum aestivum* L.) by inoculation of salinity tolerant *Azotobacter* strains. Physiol. Mol. Biol. Plants 4, 515–519. 10.1007/s12298-013-0178-2 PMC3781287 24431520

[B13] Chen Z. Sheng X. F. He L. Y. Huang Z. Zhang W. H. (2013). Effects of root inoculation with bacteria on the growth, Cd uptake and bacterial communities associated with rape grown in Cd-contaminated soil. J. Hazard. Mater., 244–245, 709–717. 10.1016/j.jhazmat.2012.10.063 23177252

[B14] Ciccillo F. Fiore A. Bevivino A. Dalmastri C. Tabacchioni S. Chiarini L. (2002). Effects of two different application methods of *Burkholderia ambifaria* MCI 7 on plant growth and rhizospheric bacterial diversity. Environ. Microbiol. 4, 238–245. 10.1046/j.1462-2920.2002.00291.x 12010130

[B15] Compant S. Mathieu F. (2013). “Use of DOPE-FISH tool to better visualize colonization of plants by beneficial bacteria? An example with *Saccharothrix algeriensis* NRRL B- 24137 colonizing grapevine plants,” in Molecular Microbial Ecology of the Rhizospher. Ed. de Bruijn F. J. (Hoboken, New Jersey: John Wiley & Sons, Ltd), 929–931. 10.1002/9781118297674.ch87

[B16] Compant S. Samad A. Faist H. Sessitch A. (2019). A review on the plant microbiome: ecology, functions, and emerging trends in microbial application. J. Adv. Res. 19, 29–37. 10.1016/j.jare.2019.03.004 31341667 PMC6630030

[B17] Couillerot O. Poirier M. A. Prigent-Combaret C. Mavingui P. Caballero-Mellado J. (2010). Assessment of SCAR markers to design real-time PCR primers for rhizosphere quantification of *Azospirillum brasilense* phytostimulatory inoculants of maize. J. Appl. Microbiol. 109, 528–538. 10.1111/j.1365-2672.2010.04673.x 20141548

[B18] Dal Cortivo C. Barion G. Visioli G. Mattarozzi M. Mosca G. (2017). Increased root growth and nitrogen accumulation in common wheat following PGPR inoculation : assessment of plant-microbe interactions by ESEM. Agric. Ecosyst. Environ. 247, 396–408. 10.1016/j.agee.2017.07.006

[B19] Daniel R. (2005). The metagenomics of soil. Nat. Rev. Microbiol. 3, 470–478. 10.1038/nrmicro1160 15931165

[B20] De Maayer P. Chan W. Y. Rubagotti E. Venter S. N. Toth. I. K. Birch P. R. J. (2014). Analysis of the *Pantoea ananatis* pan-genome reveals factors underlying its ability to colonize and interact with plant, insect and vertebrate hosts. BMC Genomics 15, 404. 10.1186/1471-2164-15-404 24884520 PMC4070556

[B21] Dennis P. G. Miller A. J. Clark I. M. Taylor R. G. Valsami-Jones E. Hirsch P. R. (2008). A novel method for sampling bacteria on plant root and soil surfaces at the microhabitat scale. J. Microbiol. Methods 75, 12–18. 10.1016/j.mimet.2008.04.013 18558444

[B22] Downie H. F. Valentine T. A. Otten W. Spiers A. J. Dupuy L. X. (2014). Transparent soil microcosms allow 3D spatial quantification of soil microbiological processes *in vivo* . Plant Signal. Behav. 9, e970421. 10.4161/15592316.2014.970421 25482802 PMC4622970

[B23] Elvang A. M. Westerberg K. Jernberg C. Jansson J. K. (2001). Use of green fluorescent protein and luciferase biomarkers to monitor survival and activity of *Arthrobacter chlorophenolicus* A6 cells during degradation of 4-chlorophenol in soil. Environ. Microbiol. 3, 32–42. 10.1046/j.1462-2920.2001.00156.x 11225721

[B24] Emerson J. B. Adams R. I. Román C. M. B. Brooks B. Coil D. A. Dahlhausen K. (2017). Schrödinger's microbes: tools for distinguishing the living from the dead in microbial ecosystems. Microbiome 5, 86. 10.1186/s40168-017-0285-3 28810907 PMC5558654

[B25] Errampalli D. Leung K. Cassidy M. B. Kostrzynska M. Blears M. Lee H. (1999). Applications of the green fluorescent protein as a molecular marker in environmental microorganisms. J. Microbiol. Methods 35, 187–199. 10.1016/S0167-7012(99)00024-X 10333070

[B26] Fan B. Chen X. H. Budiharjo A. Bleiss W. Vater J. Borriss R. (2011). Efficient colonization of plant roots by the plant growth promoting bacterium *Bacillus amyloliquefaciens* FZB42, engineered to express green fluorescent protein. J. Biotechnol. 151, 303–311. 10.1016/j.jbiotec.2010.12.022 21237217

[B27] Finkel O. M. Castrillo G. Herrera Paredes S. Salas González I. Dangl J. L. (2017). Understanding and exploiting plant beneficial microbes. Curr. Opin. Plant Biol. 38, 155–163. 10.1016/j.pbi.2017.04.018 28622659 PMC5561662

[B28] Fiorentino N. Ventorino V. Bertora C. Pepe O. Moschetti G. Grignani C. (2016). Changes in soil mineral N content and abundances of bacterial communities involved in N reactions under laboratory conditions as predictors of soil N availability to maize under field conditions. Biol. Fertil. Soils 52, 523–537. 10.1007/s00374-016-1095-7

[B29] Fiorentino N. Ventorino V. Woo S. L. Pepe O. De Rosa A. Gioia L. (2018). *Trichoderma*-based biostimulants modulate rhizosphere microbial populations and improve N uptake efficiency, yield, and nutritional quality of leafy vegetables. Front. Plant Sci. 9, 743. 10.3389/fpls.2018.00743 29922317 PMC5996573

[B30] Götz M. Gomes N. C. Dratwinski A. Costa R. Berg G. Peixoto R. (2006). Survival of *gfp*-tagged antagonistic bacteria in the rhizosphere of tomato plants and their effects on the indigenous bacterial community. FEMS Microbiol. Ecol. 56, 207–218. 10.1111/j.1574-6941.2006.00093.x 16629751

[B31] Gamalero E. Lingua G. Berta G. Lemanceau P. (2003). Methods for studying root colonization by introduced beneficial bacteria. Agronomie 23, 407–418. 10.1051/agro:2003014

[B32] Garza D. R. Dutilh B. E. (2015). From cultured to uncultured genome sequences: metagenomics and modeling microbial ecosystems. Cell Mol. Life Sci. 72, 4287–4308. 10.1007/s00018-015-2004-1 26254872 PMC4611022

[B33] Gauri S. S. Mandal S. M. Pati B. R. (2012). Impact of *Azotobacter* exopolysaccharides on sustainable agriculture. Appl. Microbiol. Biotechnol. 95, 331–338. 10.1007/s00253-012-4159-0 22615056

[B34] Germaine K. Keogh E. Garcia-Cabellos G. Borremans B. Lelie D. Barac T. O. (2004). Colonisation of poplar trees by *gfp* expressing bacterial endophytes. FEMS Microbiol. Ecol. 48, 109–118. 10.1016/j.femsec.2003.12.009 19712436

[B35] Giovannetti M. Mosse B. (1980). An evaluation of techniques for measuring vesicular arbuscular mycorrhizal infection in roots. New Phytol. 84, 489–500. 10.1111/j.1469-8137.1980.tb04556.x

[B36] Glick B. R. (2015). “Issues regarding the use of PGPB,” in Benefical plant-bacterial interactions. Ed. Glick B. R. (Switzerland: Springer International Publishing), 223–243. 10.1007/978-3-319-13921-0_8

[B37] Horvath P. Barrangou R. (2010). CRISPR/Cas, the immune system of bacteria and archaea. Science 327, 167–170. 10.1126/science.1179555 20056882

[B38] Hulse J. D. (2018). Review of comprehensive staining techniques used to differentiate arbuscular mycorrhizal fungi from plant root tissues. Acta Sci. Agric. 2, 39–44.

[B39] Jansson J. (2015). “Soil metagenomics,” in Encyclopedia of metagenomics - Environmental metagenomics. Eds. Highlander S. K. Rodriguez-Valera F. White B. A. (New York, NY: Springer), 600–609. 10.1007/978-1-4899-7475-4

[B40] Kandel S. L. Herschberger N. Kim S. H. Doty S. L. (2015). Diazotrophic endophytes of poplar and willow for growth promotion of rice plants in nitrogen-limited coditions. Crop Sci. 55, 1765–1772. 10.2135/cropsci2014.08.0570

[B41] Kandel S. L. Joubert P. M. Doty S. L. (2017). Bacterial endophyte colonization and distribution within plants. Microorganisms 5, E77. 10.3390/microorganisms5040077 29186821 PMC5748586

[B42] Kaul S. Sharma T. Dhar M. K. (2016). Omics tools for better understanding the plant-endophyte interactions. Front. Plant Sci. 7, 955. 10.3389/fpls.2016.00955 27446181 PMC4925718

[B43] Khalid M. Hassani D. Bilal M. Asad F. Huang D. (2017). Influence of bio-fertilizer containing beneficial fungi and rhizospheric bacteria on health promoting compounds and antioxidant activity of *Spinacia oleracea* L. Bot. Stud. 58, 35. 10.1186/s40529-017-0189-3 28815474 PMC5559411

[B44] Kloepper J. W. Beauchamp J. (1992). A review of issues related to measuring colonization of plant roots by bacteria. Can. J. Microbiol. 38, 1219–1232. 10.1139/m92-202

[B45] Kröber M. Wibberg D. Grosch R. Eikmeyer F. Verwaaijen B. Chowdhury S. P. (2014). Effect of the strain *Bacillus amyloliquefaciens* FZB42 on the microbial community in the rhizosphere of lettuce under field conditions analyzed by whole metagenome sequencing. Front. Microbiol. 5, 252. 10.3389/fmicb.2014.00252 24904564 PMC4033844

[B46] Krzyzanowska D. Obuchowski M. Bikowski M. Rychlowski M. Jafra S. (2012). Colonization of potato rhizosphere by GFP-tagged *Bacillus subtilis* MB73/2, *Pseudomonas* sp. P482 and *Ochrobactrum* sp. A44 shown on large sections of roots using enrichment sample preparation and confocal laser scanning microscopy. Sensors 12, 17608–17619. 10.3390/s121217608 23250280 PMC3571856

[B47] Kumar S. Suyal D. C. Bhoriyal M. Goel R. (2018). Plant growth promoting potential of psychrotolerant *Dyadobacter* sp. for pulses and finger millet and impact of inoculation on soil chemical properties and diazotrophic abundance. J. Plant Nutr. 41, 1035–1046. 10.1080/01904167.2018.1433211

[B48] Ladha J. K. Barraquio W. L. Revilla L. (1997). Isolation of endophytic diazotrophic bacteria from wetland rice. Plant Soil 194, 15–24. 10.1007/978-94-011-7113-7_3

[B49] Lang Z. Qi D. Dong J. Ren L. Zhu Q. Huang W. (2018). Isolation and characterization of a quinclorac-degrading Actinobacteria *Streptomyces* sp. strain AH-B and its implication on microecology in contaminated soil. Chemosphere 199, 210–217. 10.1016/j.chemosphere.2018.01.133 29438948

[B50] Lebeis S. L. Rott M. Dangl J. L. Schulze-Lefert P. (2012). Culturing a plant microbiome community at the cross-rhodes. New Phytol. 196, 341–344. 10.1111/j.1469-8137.2012.04336.x 22978611

[B51] Leff L. G. Leff A. A. (1996). Use of green fluorescent protein to monitor survival of genetically engineered bacteria in aquatic environments. Appl. Environ. Microbiol. 62, 3486–3488. 8795242 10.1128/aem.62.9.3486-3488.1996 PMC168148

[B52] Lima G. De Curtis F. Castoria R. De Cicco V. (2003). Integrated control of apple postharvest pathogens and survival of biocontrol yeasts in semi-commercial conditions. Eur. J. Plant Pathol. 109, 341–349. 10.1023/A:1023595529142

[B53] Lo K. J. Lin S. S. Lu C. W. Kuo C. H. Liu C. T. (2018). Whole-genome sequencing and comparative analysis of two plant-associated strains of *Rhodopseudomonas palustris* (PS3 and YSC3). Sci. Rep. 8, 12769. 10.1038/s41598-018-31128-8 30143697 PMC6109142

[B54] Lombard N. Prestat E. Van Elsas J. D. Simonet P. (2011). Soil-specific limitations for access and analysis of soil microbial communities by metagenomics. FEMS Microbiol. Ecol. 78, 31–49. 10.1111/j.1574-6941.2011.01140.x 21631545

[B55] Lopez B. R. Bashan Y. Trejo A. de-Bashan L. E. (2013). Amendment of degraded desert soil with wastewater debris containing immobilized *Chlorella sorokiniana* and *Azospirillum brasilense* significantly modifies soil bacterial community structure, diversity, and richness. Biol. Fertil. Soils 49, 1053–1063. 10.1007/s00374-013-0799-1

[B56] Lucy M. Reed E. Glick B. R. (2004). Applications of free living plant growth-promoting rhizobacteria. Antonie Van Leeuwenhoek 86, 1–25. 10.1023/B:ANTO.0000024903.10757.6e 15103234

[B57] Lusiba S. G. Odhiambo J. J. O. Ogola J. B. O. (2018). Growth, yield and water use efficiency of chickpea (*Cicer Arietinum*): response to biochar and phosphorus fertilizer application. Arch. Agron. Soil Sci. 64, 819–833. 10.1080/03650340.2017.1407027

[B58] Mardis E. R. (2008). Next-generation DNA sequencing methods. Annu. Rev. Genomics Hum. Genet. 9, 387–402. 10.1146/annurev.genom.9.081307.164359 18576944

[B59] McGhee G. C. Sundin G. W. (2012). *Erwinia amylovora* CRISPR elements provide new tools for evaluating strain diversity and for microbial source tracking. PloS One 7, e41706. 10.1371/journal.pone.0041706 22860008 PMC3409226

[B60] McInroy J. A. Kloepper J. W. (1991). Analysis of population densities and identification of endophyte bacteria of maize and cotton in the field. Plant Soil 173, 337–342. 10.1007/BF00011472

[B61] Mira A. Martín-Cuadrado A. B. D'Auria G. Rodríguez-Valera F. (2010). The bacterial pan-genome: a new paradigm in microbiology. Int. Microbiol. 13, 45–57. 10.2436/20.1501.01.110 20890839

[B62] Montella S. Ventorino V. Lombard V. Henrissat B. Pepe O. Faraco V. (2017). Discovery of genes coding for carbohydrate-active enzyme by metagenomic analysis of lignocellulosic biomasses. Sci. Rep. 15, 42623. 10.1038/srep42623 PMC5309792 28198423

[B63] Morgan J. L. Darling A. E. Eisen J. A. (2010). Metagenomic sequencing of an *in vitro*-simulated microbial community. PloS One 5, e10209. 10.1371/journal.pone.0010209 20419134 PMC2855710

[B64] Moter A. Gobel U. B. (2000). Fluorescence *in situ* hybridization (FISH) for direct visualization of microorganisms. J. Microbiol. Methods 41, 85–112. 10.1016/S0167-7012(00)00152-4 10991623

[B65] Narula N. Remus R. Deubel A. Granse A. Dudeja S. S. Behl R. K. (2007). Comparison of the effectiveness of wheat roots colonization by *Azotobacter chroococcum* and *Pantoea agglomerans* using serological techniques. Plant Soil Environ. 53, 167–176.

[B66] Neumann G. George T. S. Plassard C. (2009). Strategies and methods for studying the rhizosphere-the plant science toolbox. Plant Soil 231, 431–456. 10.1007/s11104-009-9953-9

[B67] Ngom B. Liu X. (2014). Techniques for tracking microbial community structure and function in natural environment and engineered systems. Int. J. Sci. Res. 3, 800–807.

[B68] Pantanella F. Valenti P. Natalizi T. Passeri D. Berlutti F. (2013). Analytical techniques to study microbial biofilm on abiotic surfaces: pros and cons of the main techniques currently in use. Ann. Ig. 25, 31–42. 10.7416/ai.2013.1904 23435778

[B69] Pennock D. Yates T. Braidek J. (2008). “Soil sampling designs,” in Soil sampling and methods of analysis, 2nd Edition Eds. Carter M. R. Gregorich E. G. (Boca Raton, FL: CRC Press), 1–14.

[B70] Pershina E. V. Andronov E. E. Pinaev A. G. Provorov N. A. (2013). “Recent advances and perspectives in metagenomic studies of soil microbial communities,” in Management of microbial resources in the environment. Eds. Malik A. Grohmann E. Alves M. (New York, NY: Springer), 141–166. 10.1007/978-94-007-5931-2

[B71] Phillips J. M. Hayman D. S. (1970). Improved procedures for clearing roots and staining parasitic and vesicular-arbuscular mycorrhizal fungi for rapid assessment of infection. Trans. Br. Mycol. Soc. 55, 158–161. 10.1016/S0007-1536(70)80110-3

[B72] Piromyou P. Noisangiam R. Uchiyama H. Tittabutr P. Boonkerd N. Teaumroong N. (2013). Indigenous microbial community structure in rhizosphere of Chinese kale as affected by plant growth-promoting rhizobacteria inoculation. Pedosphere 23, 577–592. 10.1016/S1002-0160(13)60051-X

[B73] Pitkäranta M. Meklin T. Hyvärinen A. Paulin L. Auvinen P. Nevalainen A. (2007). Analysis of fungal flora in indoor dust by ribosomal DNA sequence analysis, quantitative PCR, and culture. Appl. Environ. Microbiol. 74, 233–244. 10.1128/AEM.00692-07 17981947 PMC2223223

[B74] Podile A. R. Kishore K. (2006). “Plant growth-promoting rhizobacteria,” in Plant-Associated Bacteria. Ed. Gnanamanickam S. S. (Dordrecht: Springer), 195–230. 10.1007/978-1-4020-4538-7_6

[B75] Providenti M. A. Begin M. Hynes S. Lamarche C. Chitty D. Hahn J. (2009). Identification and application of AFLP-derived genetic markers for quantitative PCR-based tracking of *Bacillus* and *Paenibacillus* spp. released in soil. Can. J. Microbiol. 55, 1166–1175. 10.1016/j.plgene.2016.12.004 19935889

[B76] Quadt-Hallmann A. Kloepper J. (1996). Immunological detection and localization of the cotton endophyte *Enterobacter asburiae* JM22 in different plant species. Can. J. Microbiol. 42, 1144–1154. 10.1139/m96-146

[B77] Ribeiro C. M. Cardoso E. J. B. N. (2012). Isolation, selection and characterization of root-associated growth promoting bacteria in Brazil Pine (*Araucaria angustifolia*). Microbiol. Res. 167, 69–78. 10.1016/j.micres.2011.03.003 21596540

[B78] Richter-Heitmann T. Eickhorst T. Knauth S. Friedrich M. W. Schmidt H. (2016). Evaluation of strategies to separate root-associated microbial communities: a crucial choice in rhizobiome research. Front. Microbiol. 7, 773. 10.3389/fmicb.2016.00773 27252690 PMC4877504

[B79] Rilling J. I. Acun J. A. Nannipieri P. Cassan F. D. Maruyam F. Jorquer M. (2019). Current opinion and perspectives on the methods for tracking and monitoring plant growth‒promoting bacteria. Soil Biol. Biochem. 130, 205–219. 10.1016/j.soilbio.2018.12.012

[B80] Rothballer M. Schmid M. Hartmann A. (2003). In situ localization and PGPR-effect of *Azospirillum brasilense* strains colonizing roots of different wheat varieties. Symbiosis 34, 261–279. 10.1111/j.1574-6941.2008.00582.x

[B81] Ruppel S. Ruhlmann J. Merbach W. (2006). Quantification and localization of bacteria in plant tissues using quantitative real-time PCR and online emission fingerprinting. Plant Soil 286, 21–35. 10.1007/s11104-006-9023-5

[B82] Sharma S. D. Kumar P. Raj H. Bhardwaj S. K. (2009). Isolation of arbuscular mycorrhizal fungi and *Azotobacter chroococcum* from local litchi orchards and evaluation of their activity in the air-layers system. Sci. Hortic. 123, 117–123. 10.1016/j.scienta.2009.07.019

[B83] Sharma S. D. Kumar P. Bhardwaj S. K. Yadav S. K. (2011). Screening and selecting novel AM fungi and *Azotobacter* strain for inoculating apple under soil solarization and chemical disinfestation with mulch practices for sustainable nursery management. Sci. Hortic. 130, 164–174. 10.1016/j.scienta.2011.06.032

[B84] Sharma S. D. Sharma N. C. Sharma C. L. Kumar P. Chandel A. (2012). *Glomus*–*Azotobacter* symbiosis in apple under reduced inorganic nutrient fertilization for sustainable and economic orcharding enterprise. Sci. Hortic. 146, 175–181. 10.1016/j.scienta.2012.08.027

[B85] Sharma S. D. Kumar P. Yadav S. K. (2014). *Glomus–Azotobacter* association affects phenology of mango seedlings under reduced soil nutrient supply. Sci. Hortic. 173, 86–91. 10.1016/j.scienta.2014.04.039

[B86] Singh B. Sharma R. A. (2016). Yield enhancement of phytochemicals by *Azotobacter chroococcum* biotization in hairy roots of *Arnebia hispidissima* . Ind. Crop Prod. 81, 169–175. 10.1016/j.indcrop.2015.11.068

[B87] Singh S. R. Zargar M. Y. Najar G. R. Peer F. A. Ishaq M. (2013). Microbial dynamics, root colonization, and nutrient availability as influenced by inoculation of liquid bioinoculants in cultivars of apple seedlings. Commun. Soil Sci. Plant Anal. 44, 1511–1523. 10.1080/00103624.2012.760571

[B88] Solanki M. Garg F. C. (2014). The use of *lacZ* marker in enumeration of *Azotobacter chroococcum* in carrier based inoculants. Braz. J. Microbiol. 45 (2), 595–601. 10.1590/S1517-83822014000200030 25242946 PMC4166287

[B89] Sorek R. Kunin V. Hugenholtz P. (2008). CRISPR-a widespread system that provides acquired resistance against phages in bacteria and archaea. Nat. Rev. Microbiol. 6, 181–186. 10.1038/nrmicro1793 18157154

[B90] Sorte P. M. F. B. Simoes-Araujo J. L. de Melo L. H. V. de Souza Galisa P. Leal L. Baldani J. I. (2014). Development of a real-time PCR assay for the detection and quantification of *Gluconacetobacter diazotrophicus* in sugarcane grown under field conditions. Afr. J. Microbiol. Res. 8, 2937–2946. 10.5897/AJMR2014.6779

[B91] Stabentheiner E. Zankel A. Pölt P. (2010). Environmental scanning electron microscopy (ESEM) -a versatile tool in studying plants. Protoplasma 246, 89–99. 10.1007/s00709-010-0155-3 20446004

[B92] Stahl D. A. Kane M. D. (1992). Methods of microbial identification, tracking and monitoring of function. Curr. Opin. Biotechnol. 3, 244–252. 10.1016/0958-1669(92)90099-5

[B93] Steddom K. Menge J. A. Crowley D. Borneman J. (2002). Effect of repetititve applications of the biocontrol bacterium *Pseudomonas putida* on citrus soil microbial communities. Phytopathology 92, 857–862. 10.1094/PHYTO.2002.92.8.857 18942964

[B94] Stets M. I. Alqueres S. M. Souza E. M. Pedrosa F. D. Schmid M. Hartmann A. (2015). Quantification of *Azospirillum brasilense* FP2 bacteria in wheat roots by strain-specific quantitative PCR. Appl. Environ. Microbiol. 81, 6700–6709. 10.1128/AEM.01351-15 26187960 PMC4561692

[B95] Thokchom E. Thakuria D. Kalita M. C. Sharma C. K. Talukdar N. C. (2017). Root colonization by host-specific rhizobacteria alters indigenous root endophyte and rhizosphere soil bacterial communities and promotes the growth of mandarin orange. Eur. J. Soil Biol. 79, 48–56. 10.1016/j.ejsobi.2017.02.003

[B96] Timmusk S. Paalme V. Lagercrantz U. Nevo E. (2009). Detection and quantification of *Paenibacillus polymyxa* in the rhizosphere of wild barley (*Hordeum spontaneum*) with real-time PCR. J. Appl. Microbiol. 107, 736–745. 10.1111/j.1365-2672.2009.04265.x 19291233

[B97] Van Oosten M. J. Pepe O. De Pascale S. Silletti S. Maggio A. (2017). The role of biostimulants and bioeffectors as alleviators of abiotic stress in crop plants. Chem. Biol. Technol. Agric. 4, 5. 10.1186/s40538-017-0089-5

[B98] Van Oosten M. J. Di Stasio E. Cirillo V. Silletti S. Ventorino V. Pepe O. (2018). Root inoculation with *Azotobacter chroococcum* 76A enhances tomato plants adaptation to salt stress under low N conditions. BMC Plant Biol. 18, 205. 10.1186/s12870-018-1411-5 30236058 PMC6149061

[B99] Velmourougane K. Prasanna R. Singh S. Chawla G. Kumar A. Kumar A. S. (2017). Modulating rhizosphere colonisation, plant growth, soil nutrient availability and plant defense enzyme activity through *Trichoderma viride*- *Azotobacter chroococcum* biofilm inoculation in chickpea. Plant Soil 421, 157–174. 10.1007/s11104-017-3445-0

[B100] Ventorino V. De Marco A. Pepe O. De Santo A. V. Moschetti G. (2012). “Impact of innovative agricultural practices of carbon sequestration on soil microbial community,” in Carbon Sequestration in Agricultural Soils. A Multidisciplinary Approach to Innovative Methods. Ed. Piccolo A. (Berlin, Heidelberg: Springer), 145–177. 10.1007/978-3-642-23385-2_6

[B101] Ventorino V. Sannino F. Piccolo A. Cafaro V. Carotenuto R. Pepe O. (2014). *Methylobacterium populi* VP2: plant growth-promoting bacterium isolated from a highly polluted environment for polycyclic aromatic hydrocarbon (PAH) biodegradation. Sci. World J. 2014, 931793. 10.1155/2014/931793 PMC4135167 25152928

[B102] Ventorino V. Aliberti A. Faraco V. Robertiello A. Giacobbe S. Ercolini D. (2015). Exploring the microbiota dynamics related to vegetable biomasses degradation and study of lignocellulose-degrading bacteria for industrial biotechnological application. Sci. Rep. 5, 8161. 10.1038/srep08161 25641069 PMC4648445

[B103] Ventorino V. Pascale A. Adamo P. Rocco C. ,. N. Mori M. Faraco V. (2018). Comparative assessment of autochthonous bacterial and fungal communities and microbial biomarkers of polluted agricultural soils of the Terra dei Fuochi. Sci. Rep. 8, 14281. 10.1038/s41598-018-32688-5 30250138 PMC6155181

[B104] Ventorino V. Nicolaus B. Di Donato P. Pagliano G. Poli A. Robertiello A. (2019). Bioprospecting of exopolysaccharide −producing bacteria from different natural ecosystems for biopolymer synthesis from vinasse. Chem. Biol. Technol. Agric. 6, 18. 10.1186/s40538-019-0154-3

[B105] Vierheilig H. Coughlan A. P. Wyss U. Piché Y. (1998). Ink and vinegar, a simple staining technique for arbuscular-mycorrhizal fungi. Appl. Environ. Microbiol. 64, 5004–5007. 9835596 10.1128/aem.64.12.5004-5007.1998 PMC90956

[B106] Viscardi S. Ventorino V. Duran P. Maggio A. De Pascale S. Mora M. L. (2016). Assessment of plant growth promoting activities and abiotic stress tolerance of *Azotobacter chroococcum* strains for a potential use in sustainable agriculture. J. Soil Sci. Plant Nutr. 16, 848–863. 10.4067/S0718-95162016005000060

[B107] Wagner M. Horn M. Daims H. (2003). Fluorescence *in situ* hybridisation for the identification and characterisation of prokaryotes. Curr. Opin. Microbiol. 6, 302–309. 10.1016/S1369-5274(03)00054-7 12831908

[B108] Wang X. Zhang X. Liu L. Xiang M. Wang W. Sun X. (2015). Genomic and transcriptomic analysis of the endophytic fungus *Pestalotiopsis fici* reveals its lifestyle and high potential for synthesis of natural products. BMC Genomics 16, 28. 10.1186/s12864-014-1190-9 25623211 PMC4320822

[B109] White J. F. Torres M. S. Somu M. P. Johnson H. Irizarry I. Chen Q. (2014). Hydrogen peroxide staining to visualize intracellular bacterial infections of seedling root cells. Microsc. Res. Tech. 77, 566–573. 10.1002/jemt.22375 24825573

[B110] Wollum A. G. (1994). “Soil sampling for microbiological analysis,” in Methods of soil analysis - Part 2 - Microbiological and biochemical properties. Eds. Weaver R. W. Angle J. S. Bottomley P. S. (Madison, WI: Soil Science Society of America, Inc.), 1–14.

[B111] Woo S. L. Pepe O. (2018). Microbial consortia: promising probiotics as plant biostimulants for sustainable agriculture. Front. Plant Sci. 9, 1801. 10.3389/fpls.2018.01801 30564264 PMC6288764

[B112] Wu S. C. Cao Z. H. Li Z. G. Cheung K. C. Wong M. H. (2005). Effects of biofertilizer containing N-fixer, P and K solubilizers and AM fungi on maize growth: a greenhouse trial. Geoderma 125, 155–166. 10.1016/j.geoderma.2004.07.003

[B113] Wu C. H. Hwang Y. C. Lee W. Mulchandani A. Wood T. K. Yates M. V. (2008). Detection of recombinant *Pseudomonas putida* in the wheat rhizosphere by fluorescence *in situ* hybridization targeting mRNA and rRNA. Appl. Microbiol. Biotechnol. 79, 511–518. 10.1007/s00253-008-1438-x 18389235

